# A prospective, multi-center study of *Candida* bloodstream infections in Chile

**DOI:** 10.1371/journal.pone.0212924

**Published:** 2019-03-08

**Authors:** Maria E. Santolaya, Luis Thompson, Dona Benadof, Cecilia Tapia, Paulette Legarraga, Claudia Cortés, Marcela Rabello, Romina Valenzuela, Pamela Rojas, Ricardo Rabagliati, on behalf of the Chilean Invasive Mycosis Network

**Affiliations:** 1 Infectious Diseases Unit, Department of Pediatrics, Hospital Dr. Luis Calvo Mackenna, Faculty of Medicine, Universidad de Chile, Santiago, Chile; 2 Chilean Invasive Mycosis Network, Santiago, Chile; 3 Infectious Diseases Unit, Department of Medicine, Clínica Alemana, Universidad del Desarrollo, Santiago, Chile; 4 Microbiology Laboratory, Hospital Dr. Roberto del Río, Santiago, Chile; 5 Microbiological and Micological Program, Instituto de Ciencias Biomédicas, Faculty of Medicine, Universidad de Chile, Santiago, Chile; 6 Department of Clinical Laboratories, Faculty of Medicine, Pontificia Universidad Católica de Chile, Santiago, Chile; 7 Medicine Department, Hospital San Borja Arriarán, Clínica Santa María, Faculty of Medicine, Universidad de Chile, Santiago Chile; 8 Microbiology Laboratory, Hospital Padre Hurtado, Santiago, Chile; 9 Department of Infectious Diseases, Faculty of Medicine, Pontificia Universidad Católica de Chile, Santiago, Chile; Institute of Microbiology, SWITZERLAND

## Abstract

**Background:**

Active surveillance is necessary for improving the management and outcome of patients with candidemia. The aim of this study was to describe epidemiologic and clinical features of candidemia in children and adults in tertiary level hospitals in Chile.

**Methods:**

We conducted a prospective, multicenter, laboratory-based survey study of candidemia in 26 tertiary care hospitals in Chile, from January 2013 to October 2017.

**Results:**

A total of 780 episodes of candidemia were included, with a median incidence of 0.47/1,000 admissions. Demographic, clinical and microbiological information of 384 cases of candidemia, from 18 hospitals (7,416 beds), was included in this report. One hundred and thirty-four episodes (35%) occurred in pediatric patients and 250 (65%) in adult population. *Candida albicans* (39%), *Candida parapsilosis* (30%) and *Candida glabrata* (10%) were the leading species, with a significant difference in the distribution of species between ages. The use of central venous catheter and antibiotics were the most frequent risk factors in all age groups (> 70%). Three hundred and fifteen strains were studied for antifungal susceptibility; 21 strains (6.6%) were resistant to fluconazole, itraconazole, voriconazole, anidulafungin or micafungin. The most commonly used antifungal therapies were fluconazole (39%) and echinocandins (36%). The overall 30-day survival was 74.2%, significantly higher in infants (82%) and children (86%) compared with neonates (72%), adults (71%) and elderly (70%).

**Conclusions:**

Our prospective, multicenter surveillance study showed a low incidence of candidemia in Chile, with high 30-day survival, a large proportion of elderly patients, *C*. *glabrata* as the third most commonly identified strain, a 6.6% resistance to antifungal agents and a frequent use of echinocandins.

## Introduction

*Candida* bloodstream infection is a major cause of morbidity and mortality in hospitalized pediatric and adult patients worldwide [1–4], becoming the most common etiology of positive blood cultures in some centers in Europe and USA [5,6]. This increasing frequency is explained by the growth of populations which are at high risk of candidemia, such as immunocompromised patients, preterm neonates, elderly patients and patients admitted to Intensive Care Units (ICU) [7]. Unfortunately, along with the larger at-risk population, we still face numerous difficulties in the diagnosis, such as delayed confirmation and late start of appropriate antifungal therapy, which contribute to the high mortality rate [8, 9].

Available epidemiological information of candidemia comes largely from studies conducted in Europe and USA [9–12], with some new information from Asia and Australia [13–15]. In Latin America, a study by the Latin American Invasive Mycosis Network, which included 672 episodes of candidemia from 23 hospitals in 8 countries, identified differences compared to the epidemiology of the northern hemisphere, as higher incidence of 1.18 per 1,000 admissions, with an overall 30-day survival of 59.3%, highlighting a low survival rate of 37.7% in patients older than 60 years of age [16]. The epidemiology among pediatric patients in the region has also been described by the same group in a study including 302 patients younger than 18 years of age. Differences were observed compared to USA and Europe-based studies, with a high frequency of non-*albicans Candida* species predominating in neonates (56%) and children (64%). *Candida albicans* was the most common species in both groups (43.8% and 35.7%), followed by *Candida parapsilosis* (27.0% and 26.3%) and *Candida tropicalis* (14.6% in both groups), with a very low frequency of *Candida glabrata* (3.4% and 3.3%) [17]. Another recent prospective study from our region showed some distinctive characteristics in 158 cases of candidemia in Peru, such as a higher incidence of 2.04 per 1,000 admissions and a similar frequency of *C*. *albicans* (27.8%), *C*. *parapsilosis* (25.3%) and *C*. *tropicalis* (24.7%) [18]. Additional regional epidemiology data showed an incidence of 2.49 and 2.8 cases per 1,000 admissions in Brazil [19] and Mexico [20] and a low survival rate of 27.8% in a recent Brazilian study [21].

In Chile, epidemiological information comes from studies on fungal burden [22] and from a single-center retrospective study which described 120 episodes of candidemia in the 2000–2013 period. This study showed predominance of *C*. *albicans*, a decreasing frequency of *C*. *glabrata* and an increasing frequency of *C*. *parapsilosis* during the study period, together with an increase in the 30-day survival, up to 68.3% [23]. Nevertheless, we do not have global information about candidemia in the country and active surveillance of the epidemiological situation could be key in order to improve disease management and outcome.

In 2011, the Chilean Invasive Mycosis Network was created, whose main objectives were implementing active surveillance of different mycoses in children and adults along the country and identifying research and educational activities in order to create national guidelines for the prevention, diagnosis and treatment of invasive mycosis. The aim of this prospective, multicenter study was to describe epidemiologic and clinical features of *Candida* bloodstream infections in children and adults in tertiary level hospitals in Chile.

## Patients and methods

### Overall study design

A prospective, multicenter, laboratory-based survey observational study of candidemia was conducted from January 2013 to October 2017 in 26 tertiary care hospitals in Chile, belonging to the Chilean Invasive Mycosis Network. Of these, 18 hospitals collected epidemiological, clinical and microbiological information and 8 hospitals collected microbiological information only. This report includes information of candidemia cases from the 18 hospitals that collected epidemiological, clinical and microbiological data, except for incidence of candidemia, which was calculated considering the total number of cases included in the surveillance.

The study was approved by the local Ethics committee of each institution. In 8 of 26 hospitals (30.8%) the local ethics committee defined the need to use a signed informed consent form for patients and a signed consent form for parent/legal guardians and assent form for children older than 8 years of age. For the other 18 centers the local ethics committee considered that it was not necessary to obtain a signed patient or parent/legal guardian consent form, taking into account the observational nature of the study. In all cases, patient’s records were kept confidential and all identifiers were encoded prior to the analysis. The investigators did not participate in clinical decisions related to the patients.

The Ethics committees were: Comité de ética para la investigación en seres humanos de la Facultad de Medicina de la Universidad de Chile, Comité ético científico de la Facultad de Medicina de la Pontificia Universidad Católica, Comité de ética científico, Facultad de Medicina Clínica Alemana, Universidad del Desarrollo, Comité de ética del Servicio de Salud Metropolitano Norte, Comité de ética del Servicio de Salud Metropolitano Sur, Comité de ética del Servicio de Salud Metropolitano Oriente, Comité de ética del Servicio de Salud Metropolitano Centro, Comité de ética del Hospital San Juan de Dios, Comité de evaluación ético científico del Hospital Dr. Sótero del Río, Servicio de Salud Metropolitano Sur-Oriente, Comité de ética del Hospital Regional de Antofagasta, Comité de ética del Hospital Gustavo Fricke, Servicio de Salud Viña del Mar Quillota, Comité de ética del Hospital Dr. Hernán Henríquez Aravena, Comité de ética del Hospital Regional de Valdivia, Comité de ética del Hospital Regional de Puerto Montt.

All patients with candidemia were eligible for inclusion in the study and were followed for 30 days from the date of the diagnosis. The following clinical, demographic and laboratory data were collected in an *ad hoc* case report form, specifically designed for the study: age, gender, previous use of antibiotics, corticosteroids and antifungal drugs, previous surgery, admission to ICU, presence of cancer, neutropenia, cardiac disease, renal failure, liver disease, diabetes, use of mechanical ventilation, parenteral nutrition, dialysis, presence of a central venous catheter (CVC); clinical presentation at the time of candidemia diagnosis; treatment information and outcome. All clinical, demographic and laboratory information was collected using a web-based system. All isolates were identified at species level in the local laboratory and then sent to the reference laboratories for confirmation of species, as well as antifungal susceptibility tests.

### Microbiological identification and antifungal susceptibility

All hospitals had automated blood culture systems (either Bactec or Bac T-ALERT). Isolates were identified, at the local level, according to biochemical tests using the ID 32 C system (BioMerieux AS, Marcy l’Etoile, France). The confirmation species of *Candida* isolates was done at the reference laboratories with MALDI-TOF mass spectrometry (Bruker or Biomerieux). After identification of species, susceptibility tests were performed using a commercial broth microdilution assay (Sensititre Yeastone of Trek Diagnostic System) for amphotericin B deoxycholate, fluconazole, itraconazole, voriconazole, anidulafungin, caspofungin and micafungin, with the corresponding interpretation of the minimal inhibitory concentration, following the recommendations of the Clinical and Laboratory Standards Institute (CLSI, M27-S4) [24–26]. To define susceptibility of the strains, we used the clinical breakpoints available for *C*. *albicans*, *C*, *parapsilosis*, *Candida krusei*, *C*. *glabrata*, *C*. *tropicalis and Candida guillermondii*, according to the CLSI [24]. For the other species, the epidemiological cut off values (ECV) were used to define wild type and non-wild type strains, but not to predict therapeutic response [25,26].

### Definitions

An episode of candidemia was defined by the isolation of *Candida* spp. from one or more blood cultures. A new episode of candidemia was defined if more than 30 days had elapsed since the first positive blood culture. Persistent candidemia was defined as any positive blood culture for *Candida* species after 72 hours of antifungal therapy, without negative culture in-between. Previous surgery was defined as surgery in the last three months. Neonates were defined as patients aged ≤ 28 days, infants as patients > 28 days and ≤ 24 months, children as patients > 24 months and ≤ 18 years, adults as patients > 18 years and ≤ 60 years and the elderly as patients > 60 years of age.

### Statistical analysis

Incidence of candidemia was calculated using the number of new episodes of candidemia as numerator and the number of admissions as denominator. Categorical variables were reported as absolute and relative frequencies and compared using the Chi square test or Fisher’s exact test depending on the number of episodes per comparison group. Continuous variables were reported as medians and interquartile range (IQR) and compared according to their distribution (Student t-test or Mann-Whitney U test). Clinical, demographic and laboratory variables associated with death were identified, comparing data from surviving patients to those who died. Variables with p < 0.05 by univariate analysis were incorporated in a multivariate analysis, calculating the odds ratio (OR) with the corresponding 95% confidence interval (CI). All statistical analyses were performed using GraphPad Prism version 7.0 for Windows. P < 0.05 was considered statistically significant.

## Results

### Population characteristics

A total of 780 episodes of candidemia were collected by laboratory-based survey in 26 hospitals, belonging to the Chilean Invasive Mycosis Network, from January 2013 to October 2017. The overall incidence of candidemia was 0.47 cases per 1,000 admissions (IQR 0.25–0.78), with no significant differences between hospitals. Demographic, clinical and microbiological information from 384 cases of candidemia in 18 hospitals (7,416 beds), was included in this report. The median age of patients with candidemia was 51.5 years (IQR 7.8–66.9) and 55.2% were males. One hundred and thirty-four episodes (35%) occurred in pediatric patients (25 cases in neonates, 45 in infants and 64 in children) and 250 (65%) occurred in the adult population (102 in adults and 148 in the elderly). Median age of presentation by age group was 10 days in neonates (IQR 6–17 days), 7.1 months in infants (IQR 3.6–15.5 months), 9.6 years in children (IQR 5.1–13.3 years), 49.9 years in adults (IQR 36.9–55.3) and 70.5 years in the elderly (IQR 64.5–75.9). The median number of days of hospitalization before diagnosis of candidemia was 9 days in neonates (IQR 5–12 days), 22 days in infants (IQR 8–78 days), 7 days in children (IQR 1–18 days), 7 days in adults (IQR 1–33 days) and 9 days in the elderly (IQR 1–27 days).

### Microbiology results

Table 1 shows *Candida* species related to the age groups. In the global analysis, *C*. *albicans* was the leading agent (151 cases, 39%), followed by *C*. *parapsilosis* (114 cases, 30%), *C*. *glabrata* (37 cases, 10%) and *C*. *tropicalis* (32 cases, 8%). There were significant differences in *Candida* species between age groups. Neonates had the highest proportion of cases of *C*. *albicans* (60% compared to 39% in the overall group, *p* = 0.034), *C*. *glabrata* was highly prevalent in the adult population compared with the pediatric population (12.4% *versus* 4.5%, *p* = 0.011), while the opposite was observed for *C*. *tropicalis* (6% *versus* 13%, *p* = 0.032) and for *C*. *lusitanae* (1.2% *versus* 11.9%, *p* = 0.0001). Twenty-one of a total of 384 cases (5%) had persistent candidemia. Table 2 shows the percentage of cases with at least one additional isolation after 72 hours of antifungal therapy by age group, which ranges from 2% in adults and the elderly to 28% in neonates (*p* = 0.01).

**Table 1:** Species distribution in 384 episodes of candidemia, by age group.

Species	Neonates	Infants	Children	Adults	Elderly	Overall
	N %	N %	N %	N %	N %	N %
	25 (6.5)	45 (11.7)	64 (16.7)	102 (26.6)	148 (38.5)	384
*C*. *albicans*	15 (60)	9 (20)	26 (41)	44 (43)	57 (38)	151 (39)
*C*. *parapsilosis*	6 (24)	11 (24)	7 (11)	37 (36)	53 (36)	114 (30)
*C*. *glabrata*	0	3 (7)	3 (5)	13 (13)	18 (12)	37 (10)
*C*. *tropicalis*	0	7 (16)	10 (15)	3 (3)	12 (8)	32 (8)
*C*. *lusitaniae*	2 (8)	10 (22)	4 (6)	0	3 (2)	19 (5)
*C*. *pelliculosa*	1 (4)	4 (9)	2 (3)	1 (1)	1 (1)	9 (2.3)
*C*. *dublinensis*	0	0	3 (5)	2 (2)	2 (1)	7 (1.8)
*C*. *krusei*	0	1 (2)	3 (5)	2 (2)	1 (1)	7 (1.8)
*C*. *guillermondi*	1 (4)	0	5 (8)	0	0	6 (1.5)
*C*. *famata*	0	0	1 (1)	0	0	1 (0.3)
*C*. *inconspicua*	0	0	0	0	1 (1)	1 (0.3)

**Table 2:** Second isolation of *Candida* species after 72 hours of antifungal therapy in 384 episodes of candidemia, by age group.

	Number of episodes	Number of episodes	Percentage
		with a second isolation*	
Neonates	25	7	28
Infants	45	4	9
Children	64	5	8
Adults	102	2	2
Elderly	148	3	2
Total	384	21	5

*Second isolation was always by the same species

Three hundred and fifteen strains were studied for antifungal susceptibility. Twenty-one strains (6.6%) were resistant to one of the following antifungal drugs: fluconazole, itraconazole, voriconazole, anidulafungin or micafungin (Table 3). Regarding azoles resistance, *C*. *albicans* showed 1.6% resistance to fluconazole, but no resistance to itraconazole and voriconazole, while *C*. *parapsilosis* presented 1.1%, 1.1% and 2.2% resistance to fluconazole, itraconazole and voriconazole respectively. On the other hand, *C*. *glabrata* showed 6.6% and 20% resistance to fluconazole and itraconazole respectively, whereas *C*. *tropicalis* showed a 3.7% resistance to fluconazole, itraconazole and voriconazole. For echinocandins, *C*. *albicans* presented 0.8% resistance to anidulafungin and *C*. *glabrata* showed 10% resistance to micafungin (2 strains with MIC = 0.25μg/mL and one strain with MIC = 0.5μg/mL). Analyzing the ECV, *C*. *glabrata* presented 6.6% of strains above the ECV for amphotericin B. Regarding azoles, 48% of strains of *C*. *tropicalis* were above the ECV for fluconazole, significantly higher than was observed for *C*. *albicans* (7.8%) and *C*. *parapsilosis* (4.5%) (*p* = 0.002). We did not identify any multi-resistant strain in our study.

**Table 3:** In vitro susceptibility of *Candida* species to seven antifungal agents.

		MIC (ug/mL)						
		Ranges	MIC 50	MIC 90	SDD or I	R	Strains above ECV	total (%)
Species*	Antifungal agents				n (%)	n (%)	n (%)	
*C*. *albicans*	Amphotericin B	(0.03–1)	0.5	1			0(0)	128(47%)
	Fluconazole	(0.015–16)	0.25	1	1(0.8)	2 (1.6)	10(7.8)	
	Itraconazole	(0.008–0.5)	0.03	0.06	3(2.3)	0		
	Voriconazole	(0.008–0.25)	0.008	0.015	1(0.8)	0	5(3.9)	
	Anidulafungin	(0.015–1)	0.015	0.06	1(0.8)	1(0.8)	0(0)	
	Caspofungin	(0.008–0.25)	0.03	0.06	0	0		
	Micafungin	(0.008–0.5)	0.008	0.06	3 (2.4)	0	4(3.1)	
*C*.*parapsilosis*	Amphotericin B	(0.12–1)	0.5	1			0(0)	88(32%)
	Fluconazole	(0.03–16)	0.5	1	0	1(1.1)	4(4.5)	
	Itraconazole	(0.015–2)	0.03	0.12	1(1.1)	1(1.1)		
	Voriconazole	(0.008–8)	0.008	0.015	1(1.1)	2 (2.2)	0(0)	
	Anidulafungin	(0.015–2)	0.5	1	0	0	0(0)	
	Caspofungin	(0.008–1)	0.25	0.5	0	0		
	Micafungin	(0.015–2)	1	1	0	0	0(0)	
*C*. *glabrata*	Amphotericin B	(0.12–64)	0.5	1			2(6.6)	30(11%)
	Fluconazole	(0.5–128)	8	32	25(83.3)	2(6.6)	0	
	Itraconazole	(0.12–16)	0.5	1	20(67)	6(20)	0	
	Voriconazole	(0.06–2)	0.25	0.5			4(13.3)	
	Anidulafungin	(0.015–0.06)	0.015	0.06	0	0	0(0)	
	Caspofungin	(0.006–0.12)	0,06	0.12	0	0		
	Micafungin	(0.008–0.5)	0.015	0.06	0	3 (10)	1 (3.3)	
*C*.*tropicalis*	Amphotericin B	(0.25–1)	0.5	1			0(0)	27(10%)
	Fluconazole	(0.5–128)	2	2	1(3.7)	1(3.7)	10(48)	
	Itraconazole	(0.12–16)	0.25	0.25	13(52)	1(3.7)	1(4)	
	Voriconazole	(0.03–8)	0.12	0.25	6(2.2)	1(3.7)	10(37)	
	Anidulafungin	(0.03–0.25)	0.03	0.12	0	0	1(3.7)	
	Caspofungin	(0.06–0.12)	0.03	0.06	0	0		
	Micafungin	(0.015–0.12)	0.03	0.03	0	0	1(3.7)	

MIC = minimal inhibitory concentration; SDD = susceptible, dose-dependent; I = intermediate; R = resistant; ECV = epidemiological cutoff-value

*The total number of 273 strains included in the table represent the total number of strains of *C*. *albicans*, *C*. *parapsilosis*, *C*. *glabrata and C*. *tropicalis* studied for antifungal susceptibility.

### Risk factors

Use of CVC and use of previous antibiotics were the most frequent risk factors in all age groups, present in more than 70% of cases, followed by admission to ICU (54%), previous surgery (47%) and renal failure (45%) (Table 4). The frequency of some risk factors differed by age group. For neonates, the most common risk factors were ICU admission, mechanical ventilation and parenteral nutrition; for infants, liver disease and previous antifungal use; for children, hematological malignancies and neutropenia; for adults, renal failure and for the elderly, cardiac disease, renal failure and diabetes.

**Table 4:** Risk factors in 384 patients with candidemia, by age group.

	Neonates	Infants	Children	Adults	Elderly	Overall
	N = 25	N = 45	N = 64	N = 102	N = 148	N = 384
Variable N (%)						
Admission to ICU	24 (96)	26 (58)	31 (48)	64 (63)	62 (42)	207 (54)
Cancer	0	6 (13)	27 (42)	21 (21)	32 (22)	86 (22)
Hematology	0	3 (7)	18 (28)	9 (9)	9 (6)	39 (10)
Solid tumor	0	3 (7)	9 (14)	12 (12)	23 (16)	47 (12)
Cardiac disease	7 (28)	7 (16)	18 (28)	18 (18)	64 (43)	114 (30)
Renal failure	6 (24)	4 (9)	13 (20)	56 (55)	95 (64)	174 (45)
Liver disease	1 (4)	12 (27)	6 (9)	9 (9)	11 (7)	40 (10)
Diabetes	0	0	6 (9)	27 (27)	69 (47)	102 (27)
Mechanical ventilation	24(96)	19 (42)	20 (31)	36 (35)	34 (23)	133 (35)
Previous surgery	13 (52)	26 (58)	24 (38)	49 (48)	70 (47)	182 (47)
Parenteral nutrition	22 (88)	29 (64)	30 (47)	23 (23)	25 (17)	129 (34)
Dialysis	1 (4)	1 (2)	9 (14)	44 (43)	60 (41)	115 (30)
Neutropenia	0	5 (11)	22 (34)	9 (9)	9 (6)	45 (12)
Central venous catheter	23 (92)	44 (98)	59 (92)	78 (77)	111 (75)	315 (82)
Previous use of antibiotics	24 (96)	43 (96)	61 (95)	78 (77)	109 (74)	315 (82)
Previous use of corticosteroids	2 (8)	14 (31)	18 (28)	28 (28)	33 (22)	95 (25)
Previous use of antifungals	2 (8)	12 (27)	12 (19)	13 (12)	12 (8)	51 (13)

ICU: Intensive care unit

### Treatment

The most commonly used antifungal therapies were fluconazole (39%) and echinocandins (36%), followed by amphotericin B, which was used in 14% of patients (10% deoxicolate and 4% liposomal). The most common antifungal therapy in neonates was amphotericin B (40%), echinocandins in infants and children (56% and 47%) and fluconazole in adults and the elderly (47% and 54%). Twenty-nine out of 384 patients (8%) were not treated, 8% in neonates, 0% in infants, 2% in children, 12% in adults and 10% in the elderly, respectively (Table 5). A statistically significant difference was observed in infants and children compared to neonates, adults and the elderly (*p* = 0.001).

**Table 5:** Primary antifungal therapy of 384 patients with candidemia, by age group.

Antifungal	Neonates	Infants	Children	Adults	Elderly	Overall
	N = 25	N = 45	N = 64	N = 102	N = 148	N = 384
Deoxycolate Amphotericine B	10 (40)	5 (11)	11 (17)	8 (8)	3 (2)	37 (10)
Liposomal Amphotericine B	0	6 (13)	10 (15)	0	0	16 (4)
Echinocandins	5 (20)	25 (56)	30 (47)	32 (31)	46 (31)	138 (36)
Voriconazole	0	0	1 (2)	0	0	1
Fluconazole	6 (24)	8 (18)	9 (14)	48 (47)	81 (54)	152 (39)
Combined therapy	2 (8)	1 (2)	2 (3)	2 (2)	4 (3)	11 (3)
Untreated	2 (8)	0	1 (2)	12 (12)	14 (10)	29 (8)

### Outcome

The overall 30-day survival was 74.2%, significantly higher in infants (82%) and children (86%) compared to neonates (72%), adults (71%) and the elderly (70%) (*p* = 0.01). Survival was significantly higher in treated (77.5%) *versus* non-treated patients (34.2%) (p = 0.01). Variables associated with a high risk of mortality at day 30 using univariate analysis were admission to ICU, renal failure, mechanical ventilation, previous use of antibiotics, previous use of corticosteroids and candidemia caused by *C*. *krusei*. From these, independent predictors of 30-day mortality by multivariate analysis were mechanical ventilation (OR 2.18, 95% CI 1.25–3.78, p = 0.006) and previous use of corticosteroids (OR 2.32, 95%CI 1.33–4.07, p = 0.003).

## Discussion

Our study is, to date, the largest epidemiological report of candidemia in Chile. By including cases in children and adults, located throughout the country, it provides robust epidemiological information, which could contribute to a better understanding of candidemia in the region. Our results allowed the characterization of candidemia in Chile and identified differences with other Latin-American countries, as the lowest incidence in the region with 0.47 cases per 1,000 admissions, with predominance of *C*. *albicans*, followed by *C*. *parapsilosis* and *C*. *glabrata*. Additional differences with the Latin-American study were that cases were more frequent in the elderly (38.55 *versus* 22.6%), having twice rate of antifungal resistance (6.65 *versus* 3.1%) and higher 30-day survival (74.2% *versus* 59.3%) [16].

The incidence of 0.47 per 1,000 admissions (IQR 0.25–0.78) was similar to those reported in Australia [27], Belgium [28] and Spain [29] (0.21, 0.44 and 0.58 per 1,000 admissions, respectively) and lower than reported by Northern Ireland [30], Italy [31], Peru [18] and Brazil [19] (1.09, 1.73, 2.04, 2.49 per 1,000 admissions respectively). In a recent study that explored contributing factors for the *Candida* spp. epidemiological differences among Denmark, Finland, Norway and Sweden, results suggested that the frequency of hematological malignancies and the rate of metronidazole, colistin, piperacillin tazobactam, ciprofloxacin, carbapenems and fluconazole use could explain the differences among these countries [32]. Focused research is needed in the region to understand the cause of these differences.

*C*. *albicans* was the most frequent species identified (39%), which is consistent with observations in other continents; nevertheless, the distribution of non-*albicans* species showed differences compared to other countries. The frequency of *C*. *glabrata* is similar to the one reported in Mexico [20], Switzerland [33], USA [34] and Australia [15]. Our higher frequency of *C*. *glabrata* isolation can be explained by the greater representation of adult population in our study; in fact, in this group *C*. *glabrata* represents 12.4% *versus* 4.5% of cases among pediatric patients.

The overall antifungal resistance was 6.6%, which is double the rate found in the Latin-American study [16]. To define resistant strain, the clinical breakpoints from CLSI M27-S4 were used [24]. We observed resistance to fluconazole in all species, resistance to anidulafungin and micafungin in 0.8% and 2.4% of *C*. *albicans* strains and resistance to micafungin in 10% of *C*. *glabrata*. A recent global antifungal resistance study, which included 2,809 *Candida* isolates, collected in 29 countries worldwide, reported a fluconazole resistance of 0.4% for *C*. *albicans*, 3.8% for *C*. *parapsilosis*, 8% for *C*. *glabrata* and 2.7% for *C*. *tropicalis*, which is similar to our study [35]. The same study showed an echinocandins resistance of 0 to 5% for the most common *Candida* species, except for *C*. *parapsilosis* to anidulafungin (11.3%). Our finding of 10% resistance of *C*. *glabrata* to micafungin is similar to the alarming data of echinocandins resistance reported by other authors [26, 36]. Our results could represent a warning, suggesting the need of performing a prospective azole and echinocandins resistance surveillance in our country, reviewing the antifungal prophylaxis policies and implementing a national antifungal stewardship program before reaching a critical level of resistance [37, 38].

Despite recent international guidelines [39–42], recommending echinocandins as first line therapy, fluconazole was the most frequently used therapy, closely followed by echinocandins, with a low frequency of amphotericin B prescriptions. It is likely that continuous medical education, national and regional epidemiological data and improved access explain the growing use of echinocandins, from 6.9% in the region between 2008 and 2012 [16] to 35% in our experience. More surveillance studies are necessary to evaluate this therapeutic recommendation in the next years.

The overall and per age category 30-day survival rate was higher compared to the Latin-American study (74.2% *versus* 59.3%) [16], despite the fact that we had a higher frequency of cases among elderly patients, more identification of *C*. *glabrata* and more azole resistance. A plausible hypothesis is that in the Latin American report, 14.8% of patients with candidemia did not receive antifungal therapy, compared to 8% in our study, together with the more frequent use of echinocandins in our cases.

Our study has some limitations, including the fact that we did not regulate the policies and diagnostic procedures of each center; therefore, the diagnostic performance could have been different, resulting in heterogeneous incidence results. Another limitation is that we did not have standardized antifungal therapies for all included hospitals, which could explain the different antifungal treatment approaches of participating centers.

In conclusion, this study is an important step towards understanding the epidemiology of *Candida* blood stream infection in Latin-America, showing some specific differences in Chile, such a lower incidence, higher proportion of elderly patients, *C*. *glabrata* as the third most common strain, a warning rate of resistance, higher use of echinocandins and better survival.

## Supporting information

S1 File Candidemia, Feb 18, 2019. (XLSX)
